# The fading impact of lockdowns: A data analysis of the effectiveness of Covid-19 travel restrictions during different pandemic phases

**DOI:** 10.1371/journal.pone.0269774

**Published:** 2022-06-17

**Authors:** Barry Smyth

**Affiliations:** Insight SFI Research Centre for Data Analytics, School of Computer Science, University College Dublin, Dublin, Ireland; University of Queensland - Saint Lucia Campus: The University of Queensland, AUSTRALIA

## Abstract

As countries struggled with SARS-COV2 outbreaks at the beginning of 2021, many citizens found themselves in yet another period of increasing travel restrictions, if not a strict lockdown. At the same time there was concern that further restrictions would prove to be less effective due to a range of reasons including increasing pandemic fatigue or the lack of appropriate supports. In this study we investigate whether restrictions remained effective as a way to limit non-essential travel in order to curb virus transmission. We do this by analysing *adherence* during periods of increasing and decreasing restrictions in 125 countries during three different 4-month phases, *early* (March—June 2020), *middle* (July—October 2020), and *late* (November 2020—February 2021) over the course of the first year of the pandemic, and prior to significant population-wide vaccination. We use the strength of the relationship between restriction levels and the level of personal mobility associated with non-essential travel in order to determine the degree of adherence to the restrictions imposed. We show that there is evidence of a significant decrease in adherence to restrictions during the middle and late phases of the pandemic, compared with the early phase. Our analysis further suggests that this decrease in adherence is due to changes in mobility rather than changes in restrictions. We conclude, therefore, that restrictions have become less effective at curbing non-essential travel, which may alter the cost-benefit analysis of restrictions and lockdowns, thus highlighting the need for governments to reconsider large-scale restrictions as a containment strategy in the future, in favour of more focused or flexible mitigation approaches.

## 1 Introduction

During the first months of 2021, although an accelerating vaccine roll-out created renewed optimism in the fight against SARS-COV2, many countries continued to struggle to control new waves of infections [1–10]. Efforts were exacerbated by a combination of more transmissible variants [11–13], holiday travel, colder weather in the northern hemisphere, and a growing sense of “lockdown fatigue”. Throughout the pandemic, social distancing measures have been an important way to limit close-contacts and have proven to be effective [14–17] for controlling virus transmission. Likewise, personal mobility has been shown to be an useful proxy for social distancing [18–21]. However, the potential for *behavioural fatigue* to compromise the effectiveness of restrictions has been an important consideration in designing non-pharmaceutical interventions (NPIs) and related pandemic policies [22–25].

Although recent reports have framed increased mobility levels in terms of a gradual decrease in adherence to restrictions, the evidence that this is due to behavioural fatigue has been lacking [26]. For example, Michie et al. [27] note that surveys have shown little or no evidence of a sustained reduction in adherence to regulations and, while levels of risk and anxiety peaked during the first wave of the pandemic in March/April 2020, the motivation to adhere to government guidance and restrictions has remained high. There is evidence that financial stress can undermine support for policies that are likely to lead to income loss or unemployment [28], however. Indeed, Reicher & Drury [29] have argued that the framing of this question of adherence has been misleading, because of a focus on a rule-breaking minority rather than a resilient majority. They argue that any lower adherence rates may have less to do with waning motivation and more to do with the availability of resources. As Reicher & Drury note, this is consistent with data from early lockdowns, showing the most deprived were more likely to leave home and less likely to self-isolate, despite reporting the same motivations as the most affluent. Thus, non-adherence to regulations might be more a matter of practicality than psychology. Indeed Michie et al. [27] acknowledge substantial capability, opportunity, and motivational factors that could be contributing to lower levels of adherence, while a recent report by the World Health Organization [30] suggests governments address adherence problems by providing people with the practical advice they need to reduce their personal exposure risk [31] and live more safely with the virus.

Setting aside a discussion of the potential causes of reduced adherence, what is missing from work in this area is a large-scale assessment of whether in fact adherence to restrictions has been waning. For example, increased mobility may be due to changes in the restrictions imposed even during lockdown periods, and certainly the early lockdowns were among the most severe of the pandemic. Thus, in this work we focus on the key research question of whether there is evidence that adherence to restrictions has been fading as the pandemic has progressed. We answer this question by presenting the results of a large-scale data analysis of the strength of the relationship between restrictions and non-essential travel, as a proxy for the public’s adherence to restrictions, and to assess how this has changed during the course of the pandemic, as restrictions have been imposed and relaxed. To do this we use a dataset of 125 countries with restriction and mobility data from March 2020 to the end of February 2021; this provides a full year of data prior to significant population-wide vaccination. We measure *adherence* by evaluating the strength of the relationship between restrictions and non-essential travel using two different measures. We separately measure adherence during periods of *increasing* and *decreasing* restrictions and compare average adherence levels during three 4-month phases, *early* (March—June 2020), *middle* (July—October 2020), and *late* (November 2020—February 2021), to determine whether there has been any evidence of a change in adherence.

A key finding of this study is that there is statistically reliable evidence of greater adherence to restrictions during periods of increasing restrictions, compared with periods of decreasing restrictions, however adherence has been falling for both increasing and decreasing periods as the pandemic has unfolded. There is also evidence that this decrease in adherence has been due to changes in mobility rather than changes in restrictions. We conclude, therefore, that restrictions have become less effective at curbing non-essential travel, which may alter the cost-benefit calculus of lockdowns. This is an important finding given that the economic, social, and secondary-health costs of prolonged periods of restrictions are so high [32]. If restrictions are becoming less effective then this will necessarily change their value and it will be incumbent on governments to reconsider whether restrictions, which have worked in the past, will continue to be effective in the future, and to base future policy on a robust understanding of any change in their potential.

## 2 Materials and methods

### 2.1 Data sources

The restrictions dataset [5] used is based on a categorisation of government responses to the pandemic (containment, economic, health, and other measures). Each measure is normalised between 0 and 100 and for this work we calculate a mean *travel restriction index* from a subset of measures: school and workplace closings, restrictions on gatherings and public events, and limits on public transport and personal movement.

The mobility dataset used is comprised of publicly available Google mobility data [33] for 134 countries based on daily changes in mobility relative to pre-pandemic levels. We calculate an average *mobility drop* for *non-essential travel* based on retail and recreation, public transport, and workplace related mobility categories. It is acknowledged that this unweighted average is a limitation of the work, and one that could be further explored in the future to consider alternative category weightings. This is discussed further in the discussion section later in this article.

These daily restriction and mobility data are smoothed using a 7-day rolling average and the resulting dataset includes both restriction and mobility data for a subset of 125 countries from March 1, 2020 to February 28, 2021. These countries were selected from a larger subset because (a) they were also represented in the restrictions dataset and (b) they were sufficiently well covered by Google’s mobility data; not every country is well covered by Google’s mobility reports due to missing mobility categories or missing days. This combined dataset is available as part of the supplementary material accompanying this work.

### 2.2 Evaluating the strength of the relationship between restrictions and mobility

The main objective of this work is to evaluate the strength of the relationship between restrictions and the mobility drop over time; that is, we wish to measure the degree to which changes in the levels of travel restrictions impact non-essential travel. We do this using two different *adherence* metrics, as follows:

1. *Coefficient of Determination* (*r*^2^)—we calculate *r*, Pearson’s correlation coefficient, between daily restrictions (*p*_*r*_) and the mobility drop (*p*_*m*_) for a given period of time, *p*. Then, *r*^2^ is the coefficient of determination, which is a common metric to judge the strength of the relationship between two sets of values. To allow for anticipatory or delayed mobility effects, as restrictions change, *r*^2^ is calculated using a cross-correlation technique to identify the lag, measured in days, −7 ≤ *lag*(*p*) < 7, which leads to the maximum correlation coefficient for a given set of restriction and mobility data, as shown in Eqs 1 and 2. In other words, we use the maximum *r*^2^ found by shifting the daily mobility data by up to plus/minus one week from a change in restrictions.
2. *Dynamic Time Warping Similarity* (*DTW*)—as an alternative approach, we also calculate the similarity between the daily restriction levels of a period and the corresponding mobility drops. To do this we first convert the restriction and mobility data for the period into *z* scores and then apply *dynamic time warping*[34] to calculate the distance between these data for the period; see Eq 3. Dynamic time warping is used because it provides a principled way to identify the optimal match between two time-series by non-linearly warping the two series. In this way we can also account for anticipatory or delayed mobility effects. The resulting *distance* measure is converted into a *similarity* measure (*DTW Sim*) using a *min-max* transformation, so that high similarity values indicate closer matches between restrictions and mobility.

Note, that in the first metric we use Pearson’s correlation coefficient in order to assess the linear relationship between restrictions and mobility. Alternatively a rank correlation metric such as Spearman’s correlation coefficient could be used but it will provide an assessment of rank correlation only. We discuss this further in Section 4.

$$\begin{array}{c} lag\left(p\right)=\underset{-7\;<\;d\;<\;7}{\text{arg}\;\text{max}}\;corr(p_{r},shift\left(p_{m},d\right)), \end{array}$$ (1)

$$\begin{array}{c} r^{2}\left(p\right)=corr\;{\left(p_{r},shift(p_{m},lag\left(p\right)\right)}^{2}, \end{array}$$ (2)

$$\begin{array}{c} DTW\left(p\right)=dtw(zscores(r),zscores(m)), \end{array}$$ (3)

### 2.3 Comparing increasing and decreasing periods

An important question in this work concerns whether people modulate their mobility differently during extended periods of increasing and decreasing restrictions. Obviously we can expect mobility levels to increase when restrictions are falling, and vice versa when restrictions are rising, but is the strength of the relationship between restrictions and mobility similar during periods of increasing and decreasing restrictions? To test this, the raw (unsmoothed) restrictions data are used to identify consecutive periods of *increasing* and *decreasing* restrictions for each country.

An *increasing period* begins on the first day that restrictions start to increase and continues until such time as restrictions start to decrease, thereby marking the beginning of a new decreasing period. Likewise, a *decreasing period* begins on the first day that restrictions start to fall and continues until they start to rise again. Thus, consecutive days of increasing (non-decreasing) or decreasing (non-increasing) restrictions are identified as mutually exclusive increasing or decreasing periods, respectively; a minimum period length of 14 days is used to exclude short-term or localised reversals in restriction levels from our analysis. Then, for each increasing and decreasing period, *p*, we estimate the strength of the relationship between its restrictions and mobility data using the *r*^2^ and *DTW* metrics, and we also calculate the mean restrictions and mobility drop for the period.

Since we are interested in how adherence changes over time we analyse the effect of the timing of restrictions by dividing the pandemic into three, equal, four-month *phases*: *early* (March—June 2020), *middle* (July—October 2020), and *late* (November 2020—February 2021). It is important to note that in doing so, it is *not* the case that we are assuming that the pandemic experience will be similar across all countries and phases. Clearly, different countries have been impacted by the pandemic at different times, and this will be reflected in the country-level increasing and decreasing periods of restrictions. For example, a given period of restrictions, whether increasing or decreasing, may extend from one pandemic phase into the next and in very rare cases some countries have not significantly modulated their restrictions for extended periods of time; for example, later we will note how Belarus is an outlier in this regard, because it only implemented a single short period of increasing restrictions during the early phase of the pandemic. The purpose of the three phases is to provide a reasonable way to compare country-specific adherence during early, middle, and late stages of the pandemic. In our analysis we calculate the average restriction level, mobility drop, and adherence values for increasing and decreasing periods, overall and during each pandemic phase.

### 2.4 Statistical analysis

To determine whether there is an difference in the overall strength of the relationship between restrictions and mobility, during the period from March 2020 to February 2021, we use a Wilcoxon Signed Rank Test to compare *r*^2^ and *DTW* measures for each country during increasing and decreasing periods of restrictions. A Wilcoxon Signed Rank Test was necessary because the *r*^2^, and *DTW* data are not normally distributed.

Next, to compare how adherence changes across the pandemic phases we use a Kruskal Wallis test to determine whether the *phase* of restrictions has a statistically significant effect on the various adherence measures, followed by a post hoc Dunn’s test to identify which specific pairs of phases, if any, were significantly different; Kruskal Wallis and Dunn’s tests were used because of the non-normal nature of the adherence data.

We use a similar approach to compare the mean level of restrictions and mean mobility drop during early, middle, and late phases, although in the case of the mobility drop we use a one-way ANOVA followed by a Tukey HSD test, because the mobility data is normally distributed. In all statistical tests we used *p* = 0.05 as the threshold for statistical significance.

## 3 Results

**Fig 1:**
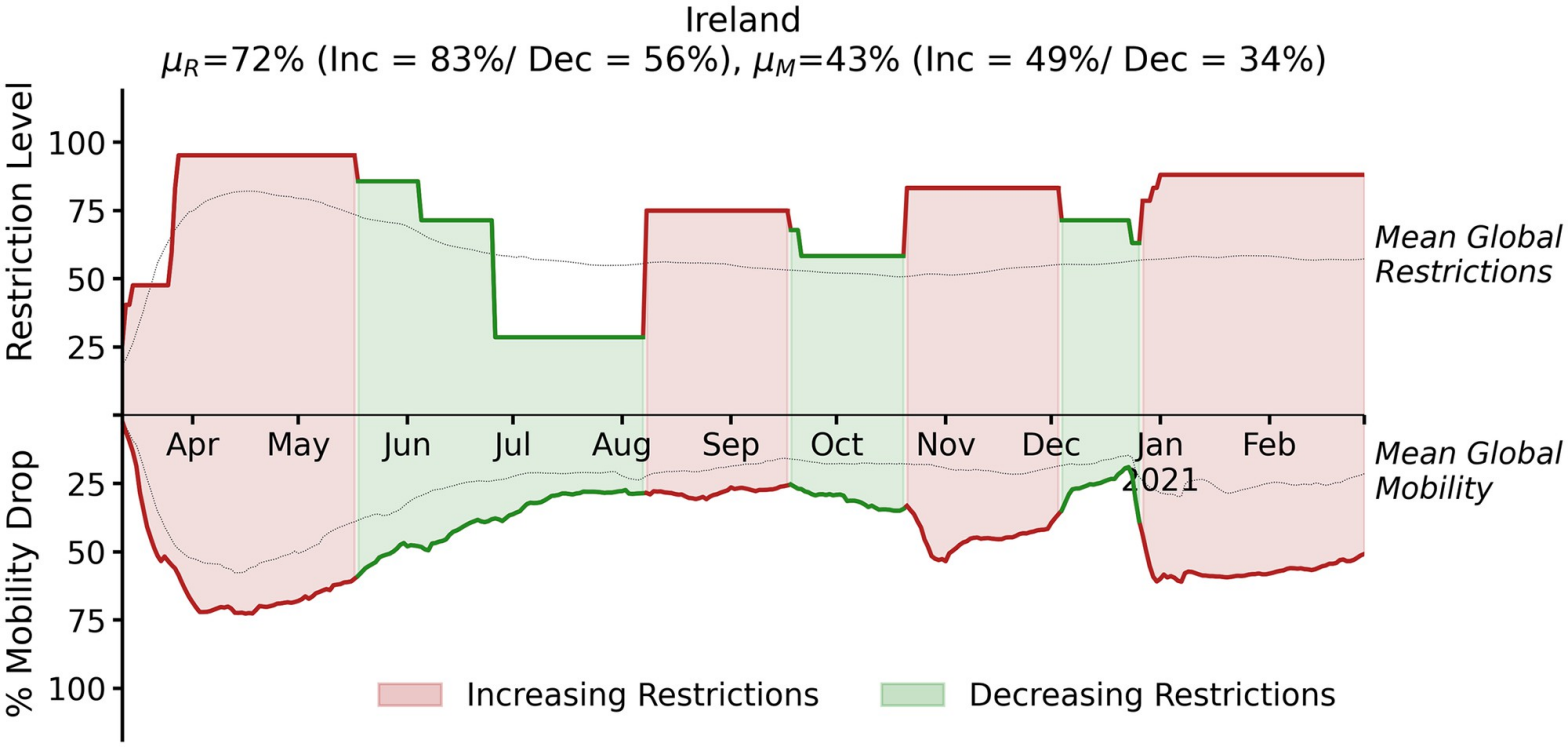
The travel-related restriction (upper graph) and mobility data (lower graph) for Ireland (7d rolling averages), from March 2020 to February 2021, inclusive. The mean global restrictions and mobility drops are also indicated as separate line graphs. The title of the graph shows the overall mean level of restrictions for Ireland (*μ*_*R*_) with the means for increasing (*Inc*) and decreasing (*Dec*) periods indicated in brackets. Similarly, the mean mobility drop for Ireland (*μ*_*M*_) and the corresponding means for the increasing and decreasing periods are also shown in brackets.

An example of the identification of periods of increasing and decreasing restrictions, and the corresponding impact on mobility, is shown in Fig 1 for Ireland. We can see how Ireland’s restrictions have mostly been higher, and its mobility drops greater, than the global averages shown.

**Fig 2:**
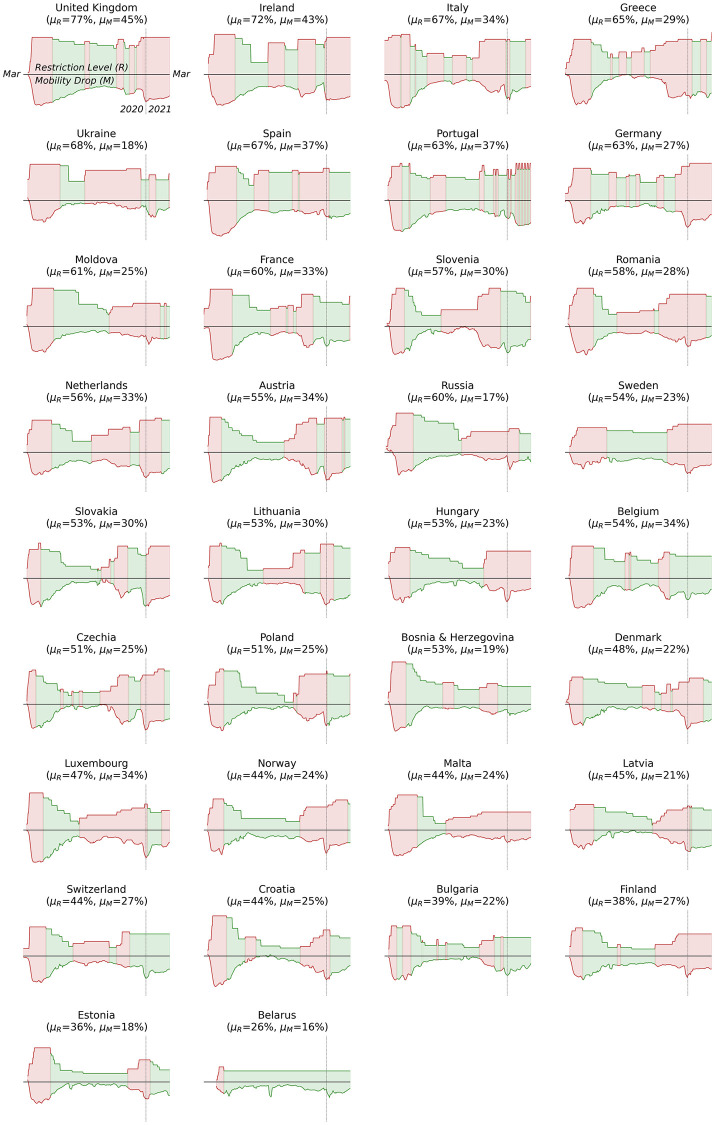
The travel-related restrictions and mobility data for 34 countries in Europe, showing periods of increasing (red) and decreasing (green) restrictions. For each country the upper graph shows the restriction level and the lower graph shows the corresponding mobility drop. Each country title also includes the mean level of restriction (*μ*_*R*_) and mobility drop (*μ*_*M*_) experienced between March 2020 and the end of February 2021.

For reference Fig 2 shows a *‘small multiples’* grid of similar graphs for 34 countries in Europe, in decreasing order of mean restrictions (*μ*_*R*_) for the period of study. They are provided here to illustrate the different approaches that have been adopted, even within a single economic/geographic region of the world. For example, we can see how most countries have cycled through multiple periods of increasing and decreasing restrictions during the first 12 months of the pandemic. Ireland and the UK stand-out somewhat, because of higher levels of restrictions than most (and higher mobility drops). Many others saw a more significant and sustained easing of restrictions during the summer of 2020, followed by a return to increased restrictions during the autumn and winter months (see for example, Greece, Austria, Germany, Slovakia and others); for these countries we can see a characteristic (rotated) hour-glass shape as the difference between restrictions and mobility drops narrow between two periods of widening. In contrast, some countries (e.g. Croatia, Bulgaria, Estonia, and others have chosen to limit restrictions during the second half of the pandemic to a much greater extent than others with Belarus standing out as having implemented a very short period of increasing restrictions during the early phase of the pandemic only.

### 3.1 Comparing increasing and decreasing periods

The results of the Wilcoxon Signed Rank Test in Table 1 indicate that periods of increasing restrictions are associated with greater levels of adherence than periods of decreasing restrictions (*p* < 0.001 for each adherence metric) across the 12 month period of this study. This suggests that people may be modulating their mobility more effectively when restrictions are increasing than when they are decreasing. In other words, people appear to be changing their mobility levels in a manner that better reflects changing levels of restrictions during increasing periods than during decreasing periods. Note, this does not necessarily mean that people regain their mobility faster during decreasing periods. It could just as easily mean that people are more cautious when restriction levels fall, and that they are slower to regain their mobility than the current restriction level might suggest, for example.

**Table 1:** The results of the Wicoxon Signed Rank Test comparing increasing and decreasing periods of restrictions for each adherence metric across the 12 month period from March 2020 to the end of February 2021.

Metric	Median(Increasing)	Median(Decreasing)	Z	p
*r* ^2^	0.61	0.51	2685.0	<0.001
DTW	0.40	0.33	2561.0	<0.001

### 3.2 Restrictions and mobility during pandemic phases

**Fig 3:**
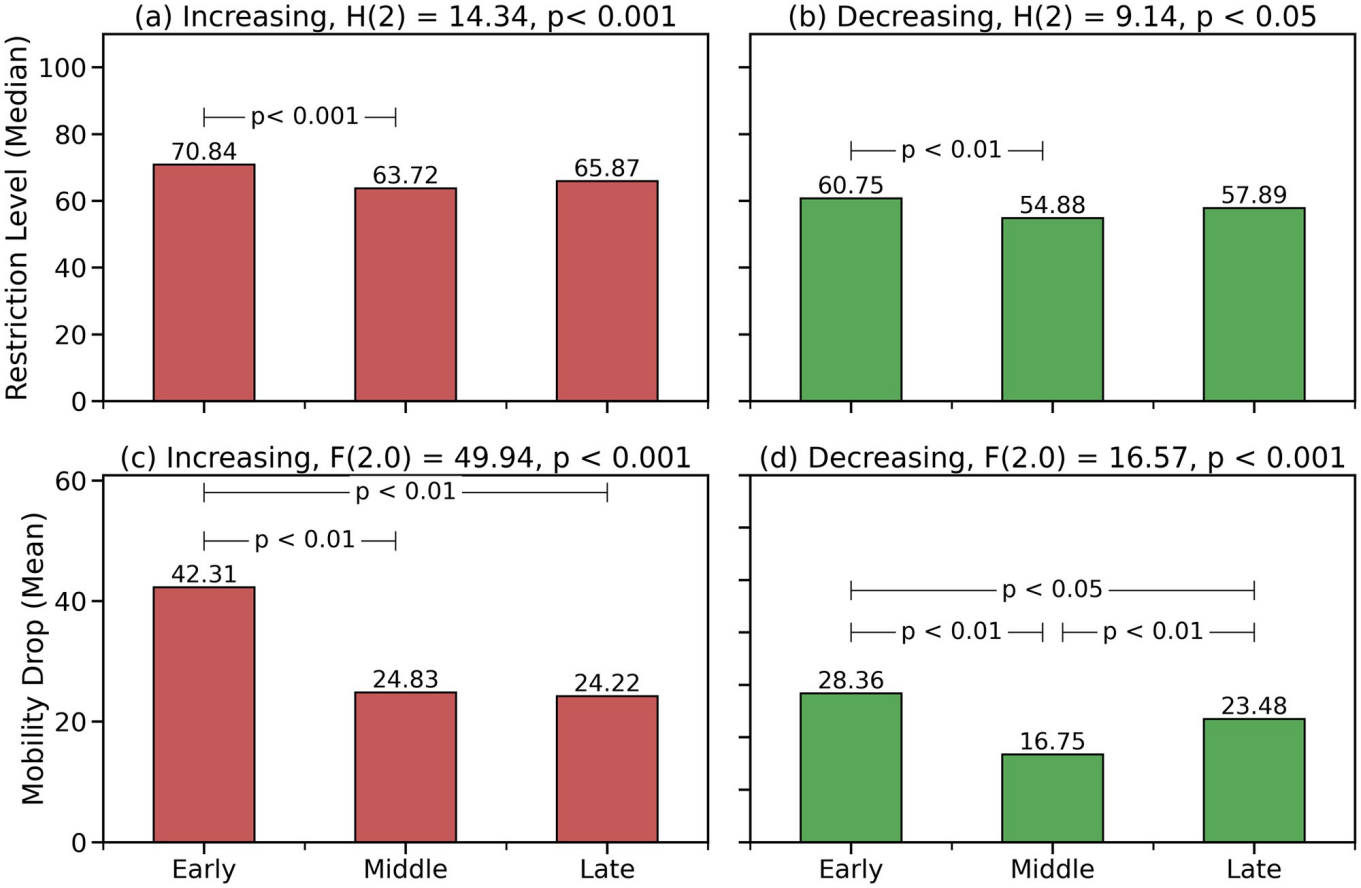
The results of a Kruskal Wallis test comparing restriction levels, during increasing and decreasing periods, by pandemic phase are shown as the title of each graph in (a) and (b): the *H* and *p* values are provided along with degrees of freedom for each*H* value in brackets as is the common practice. The *p* values for the subsequent post hoc Dunn’s test are shown in (a) and (b) as horizontal lines connecting the phases for which there is a significant difference in restriction level with the corresponding *p* value threshold shown. Similarly, the results of the one-way ANOVA and Tukey HSD tests are shown in (c) and (d) for mobility drops with the *F* and *p* values for the one-way ANOVA indicated in the titles of (c) and (d) and the subsequent Tukey HSD results for significant pairs shown in the corresponding bar graph.

Fig 3 shows the results of the analysis of restriction levels and mobility drops by pandemic phase. For example, in Fig 3(a) we see that during increasing periods the median level of restrictions was just over 70% in the early pandemic phase before dropping to just over 63% and 65% for the middle and late phases, respectively. Likewise, in Fig 3(c) we can see how the mean mobility drop was just over 42% during the early phase before falling to just over 24% in the middle and late phases.

The Kruskal Wallis results in Fig 3(a) & 3(b) show that the phase of the pandemic is associated with a significant effect on median restriction level. Note, the title text in Fig 3(a) & 3(b) shows the Kruskal Wallis results with *p* values less than 0.001 and 0.05, for increasing and decreasing periods, respectively, indicating that the pandemic phase does have a significant effect on restriction level, but not which pairs of phases are statistically different. The subsequent Dunn’s tests confirms that significance is limited to the median level of restrictions between the early and middle phases only, for both increasing and decreasing restrictions; the *p* values associated with statistically significant differences are shown in Fig 3(a) & 3(b) with horizontal bars used to indicate the relevant pandemic phases. In other words, in Fig 3(a) & 3(b) there is only a significant difference in the average level of restrictions between the early and middle phases of the pandemic; *p* < 0.001 for increasing periods and *p* < 0.01 for decreasing periods.

In contrast, the corresponding one-way ANOVA and Tukey HSD results for the normally distributed mobility data, in Fig 3(c) & 3(d), show a significant decrease in the mean mobility drop during both the middle phases (*p* < 0.01 for increasing and decreasing periods) and late phases (*p* < 0.01 for increasing and *p* < 0.05 for decreasing periods) of the pandemic, compared with the early phase; once again the title text of each graph shows the results of the ANOVA with the *p* values of the subsequent Tukey test indicated with horizontal bars in an exactly analogous manner to Fig 3(a) & 3(b). Additionally, there is also a significant difference (*p* < 0.01) in the mean mobility drop in decreasing periods between the middle and late phases of the pandemic, as shown in Fig 3(d); the mean mobility drop for decreasing periods in the late phase of the pandemic was less than the early phase but greater than the middle phase. This difference is absent from the increasing periods in Fig 3(c) where the mean mobility drop for the late phase is not statistically different from the middle phase.

Thus, there is evidence that mobility levels have been falling less after the early phase of the pandemic, especially during increasing periods of restrictions, despite somewhat similar levels of restrictions being imposed. This is already a sign that restrictions have become less effective, but on it’s own does not tell us about the change in the strength of the relationship between restrictions and mobility; for example, absolute mobility may have dropped but the level of adherence—the strength of the relationship between restrictions and mobility—may remain stable.

### 3.3 Adherence to restrictions during pandemic phases

**Fig 4:**
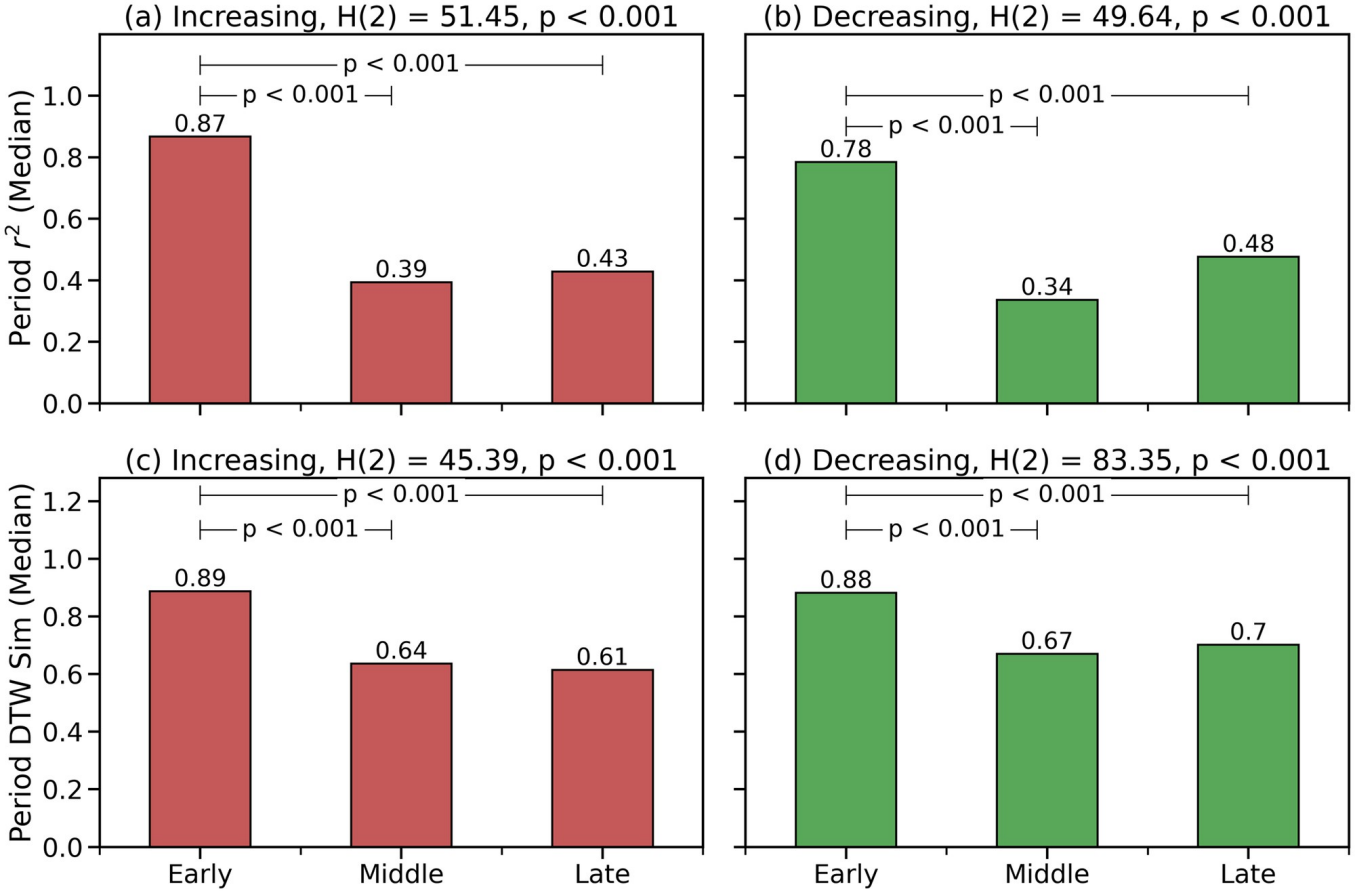
The results of a Kruskal Wallis test comparing the various adherence metrics, during increasing and decreasing periods, by pandemic phase are shown as the title of each graph: The *H* and *p* values are provided along with the degrees of freedom shown for each *H* value in brackets. The *p* values for the subsequent post hoc Dunn’s test are shown as horizontal lines connecting the periods for which there is a significant difference in adherence metric value, with the corresponding *p* value threshold indicated.

Fig 4 shows the two different measures of adherence, for increasing and decreasing periods, during the three pandemic phases. For example, in Fig 4(a) we can see a high *r*^2^ value of 0.87 for increasing periods during the early phase of the pandemic, indicating that 87% of the variation in mobility drop can be explained by the variation in restriction levels. However, this falls to 0.39 during the middle phase before increasing marginally to 0.43 for the late phase; there is a similar pattern in evidence for the DTW metric and for decreasing periods too.

Fig 4 shows the corresponding Kurskal Wallis and post hoc Dunn’s results for each of the adherence metrics; once again, the title text of the graphs shows the Kurskal Wallis results while the subsequent Dunn’s results are indicated within each bar chart using horizontal lines to indicate the relevant statistically significant differences between pairs of phases. The results for increasing periods—Fig 4(a) & 4(c)—are similar for both adherence metrics, showing that the decrease in adherence during the middle and late phases is statistically significant compared with the early phase of the pandemic (*p* < 0.001). Notably, the modest differences in adherence between the middle and late phases are not statistically significant. The results for decreasing periods—Fig 4(b) & 4(d)—are very similar to those for increasing periods.

## 4 Discussion

The main finding of this work is that the strength of the relationship between restrictions and mobility has been fading during the middle and late phases of the pandemic, especially during periods of increasing restrictions; a similar pattern of results is evident for both adherence metrics. This means that populations have been responding to restrictions in a less predictable fashion later in the pandemic. During the early phase of the pandemic, mobility levels were suppressed more than they were in later phases, even allowing for the levels of restrictions imposed.

While this points to a shift in pandemic behaviour it does not explain the root cause of this shift, which began in the summer of 2020—after the first-wave of lockdowns and long before any vaccines were available—and which persisted through to the end of February 2021. It is reasonable to conclude that this will alter the cost-benefit calculus of restrictions and the results provide an important basis to inform new questions about whether similar restrictions and lockdowns should be, or can be, relied upon later in the pandemic. At the very least it suggests that the type of restrictions that proved to be reasonably successful during the early phase of the pandemic can no longer be relied upon to curb transmission as effectively as they once did. And given the high socioeconomic burden of lockdowns and restrictions this raises real doubts about their likely practical utility going forward.

### 4.1 Shifting pandemic behaviour

As mentioned previously, Michie et al. [27] have argued that there is no strong evidence of pandemic fatigue per se, because levels of anxiety have remained high throughout the pandemic, while acknowledging that there is some evidence that changes in adherence may be linked to the financial hardship imposed by restrictions; see also [28, 29]. Nevertheless, our findings show that globally the effect of restrictions has been weakening as more people are moving about more for a given level of restrictions. Recently, Gelfand et al. [35] have highlighted the importance of social norms—or cultural tightness-looseness [36]—in the experiences of countries during the pandemic. They showed that countries with high levels of cultural tightness (strict social norms, strong punishments for defiance etc.) had far fewer cases and far fewer deaths, than countries with lower levels of cultural tightness, taking into account a number of controls. Their work suggests that tighter groups have a tendency to cooperate faster and more effectively in response to threats, compared with looser groups. A worthwhile future study will be to evaluate whether cultural tightness is also a factor in the degree of adherence to restrictions, although it will be important to control for the level of restrictions imposed.

Of course, as vaccination rates continue to grow, restrictions may prove to be less effective or acceptable. For instance, vaccinated groups may come to rely on their positive vaccination status as their primary mode of protection, notwithstanding the potential for breakthrough infections, while an unvaccinated minority may feel protected by the vaccinated majority. The availability of new therapies [37] may similarly change the perceived risk for many. Using the approach taken in this study it may be feasible to, for example, separately analyse the adherence rates of vaccinated and unvaccinated groups to better understand these aspects of behaviour. Indeed, the methodology adopted here may prove to be useful for a variety of future studies to evaluate the impact of more precisely targeted restrictions, in order to inform and optimise future mitigation strategies. And by providing governments with the ability to better evaluate near real-time adherence to restrictions, and related advisories, this work can help to inform future policy-making, both for the present pandemic and potentially for any future, as yet unknown, public health crises.

### 4.2 Limitations

It is worth highlighting a number of limitations of this work. First, and foremost it relies on Google mobility data, which cannot be guaranteed to provide a complete account of mobility levels, because it depends on the usage of Google devices in a given country. For example, there are many countries that are either absent from Google’s mobility reports or with incomplete data. These countries have been excluded from this analysis. Another limitation of the mobility data is the simple averaging used to combine the mobility associated with non-essential travel in terms of retail and recreation, public transport, and workplace related categories. An argument could be made in favour of a weighted average by using different weights for these different mobility categories. The decision to use a simple, unweighted average in this work was made on that basis that if such a simple average demonstrated an effect then a more sophisticated analysis may show an even greater effect. We did find that this simple average was sufficient to demonstrate a decrease in the level of adherence to restrictions and suggest that an exploration of alternative category weightings may be a fruitful line of future research.

A further limitation concerns the definition of the three pandemic phases (early, middle, and late). These were defined based on a simple partitioning of the pandemic into three equal (4-month phases), which broadly aligned with different pandemic waves. While this straightforward approach did serve to demonstrate a decrease in the level of adherence to restrictions, it may also be worthwhile to consider different ways to partition the pandemic, perhaps at a more regional or country-specific level. Indeed in larger countries, such as the US, very different policies were in place at different times across different states, for example. This extension to the present analysis is left as a matter for future work.

Finally, in this work we focused on two particular ways to evaluate the strength of the relationship between mobility and restrictions by using a correlation-based measure and a similarity-based measure (dynamic time warping). As noted earlier, Pearson’s correlation coefficient was for the former, primarily, because we are interested in whether there is a linear relationship between mobility and restrictions. During the course of this study we also considered the use of a rank correlation metric, specifically Spearman’s correlation coefficient and found broadly similar results. Nevertheless, it is a useful matter for future work to consider alternative ways to measure the strength of the relationship between mobility and restrictions, beyond the two measures used in this work.

## 5 Conclusions

We conducted a large-scale data analysis of COVID restrictions and mobility patterns in 125 countries, during the 12 month period from March 2020, to evaluate the effectiveness of travel-related restrictions in terms of their ability to modulate non-essential travel. The results indicate that the strength of the relationship between restrictions and mobility has been higher during periods of increasing restrictions compared with periods of decreasing restrictions. However, the strength of this relationship has been falling, for increasing and decreasing periods, during period of study.

These results indicate a reduction in the utility of restrictions, as way to limit non-essential travel, and suggest that new approaches will be needed in the future. Even with wide-scale vaccine rollouts, it is likely that virus transmission will need to be actively contained for some time to come, depending on regional variations in vaccination coverage, the longevity of immunity, they availability of effective therapies, and whether variants evolve to escape the vaccines. Thus, it is likely that governments will need to change their approach to restrictions, perhaps by using much more proactive and more targeted measures, by detecting outbreaks sooner, and identifying the original source of infections more reliably. The results of this study, and follow-on studies, should help to inform governments and public health officials about the ongoing effectiveness of restrictions and so help to guide future mitigation approaches and policy making.

## Supporting information

S1 Dataset Consolidated dataset. The full consolidated dataset used in this study is provided as a comma-separated file and based on original data provided by Our World in Data, Google, and the Blavatnik School of Government, Oxford University.
(CSV)

S1 Code Analysis source code. A Jupyter notebook (Python) is provided as part of the supporting information with the code required to reproduce the analysis presented and to generate the graphs and results included in this study. Necessary requirements and dependencies to execute this code are included in the Jupyter notebook file.
(IPYNB)

S1 File (DS_STORE)
